# Development of a Tailored Online Video-Based Assistant to Support Prenatal Screening Decisions in Couples With Limited Health Literacy: User-Centered Design Approach

**DOI:** 10.2196/75391

**Published:** 2026-03-27

**Authors:** Katharina Preuhs, Hilde van Keulen, Jeroen Pronk, Marlies Rijnders, Angelique Wils, Marianne Nieuwenhuijze, Naïma Abouri, Pepijn van Empelen

**Affiliations:** 1 Expertise Group Child Health Netherlands Organisation for Applied Scientific Research Leiden The Netherlands; 2 Academie Verloskunde Maastricht Zuyd University Maastricht, Limburg The Netherlands; 3 CAPHRI Maastricht University Maastricht, Limburg The Netherlands; 4 Pharos Utrecht The Netherlands; 5 Department of Health, Medical and Neuropsychology Leiden University Leiden, South Holland The Netherlands

**Keywords:** tailored video intervention, shared decision-making, informed decision-making, prenatal screening, health literacy, counseling aid, decision aid, vulnerable individuals

## Abstract

**Background:**

Up to 25% of pregnant couples in the Netherlands do not make an informed decision about prenatal screening: their decisions are value-inconsistent or based on insufficient knowledge and deliberation. More than one-third (36%) of the population in the Netherlands has limited health literacy skills, with the majority being individuals with lower levels of education or a migration background. They experience serious problems in understanding health information and taking an active role in decision-making. Therefore, the Dutch Health Council recommends improving decision support for pregnant couples.

**Objective:**

This study aimed to describe the rationale and systematic design of an online, interactive, and tailored video-based assistant to support pregnant couples with limited health literacy skills in decision-making on prenatal screening.

**Methods:**

The intervention mapping framework was used for the iterative user-centered development of the decision aid prenatal screening. This includes the following steps: (1) a needs assessment among the target group (ie, pregnant couples and counselors), (2) defining change objectives based on the needs assessment, (3) selection of theoretical methods, (4) program production and prototype testing, (5) implementation planning, and (6) preparation for evaluation. Three prototypes of the decision aid were iteratively tested among pregnant couples (with low literacy), counselors, and stakeholders relevant for future implementation. This paper describes steps 1 to 4 of the decision aid development.

**Results:**

We developed a decision aid guided by a virtual assistant to promote informed decision-making among pregnant couples (with low health literacy) on prenatal screening. To comply with users’ needs, it includes the following four interactive modules: (1) Other people’s experiences, (2) Information about the tests (and anomalies), (3) Help me decide, and (4) Further questions. To increase accessibility, it features a menu that allows for adapting the speed of speech, making use of subtitles, and is offered in 3 different languages. To increase implementation and future use of the decision aid, an e-learning tool was developed. The decision aid can be used either as a stand-alone tool by pregnant couples or in combination with a counselor during counseling sessions.

**Conclusions:**

By describing the systematic development of a prenatal screening decision aid designed to support pregnant couples with low health literacy in making well-informed choices, we aimed to contribute to systematic reporting and transparent intervention design.

**Trial Registration:**

International Standard Randomised Controlled Trial Registry ISRCTN18016226; https://www.isrctn.com/ISRCTN18016226

## Introduction

Prenatal screening is routinely offered in the first trimester to support prospective parents in autonomous reproductive decision-making. These screenings empower pregnant couples to make informed decisions about preparing for a child with anomalies or considering pregnancy termination. In the Netherlands, two types of prenatal screening are offered: (1) a noninvasive prenatal testing (NIPT), which is a blood test available from 10 weeks of pregnancy that screens for chromosomal anomalies, such as Down syndrome, Edwards’ syndrome, and Patau syndrome. This test may also reveal incidental findings. (2) Ultrasound scans at 13 and 20 weeks to screen for structural fetal anomalies [1]. The screening is centrally regulated via the Dutch Center for Population Screening. Unique to the Dutch program is the training provided to counselors in nondirective, value-sensitive counseling, enabling prospective parents to make decisions aligned with their own norms and values. In contrast to other Western countries—where prenatal screening is either not offered on a nationwide level (including Germany, Spain, or England) or is offered only to women at intermediate to high risk based on combined first-trimester screening (including France, Italy, and Sweden)—the Netherlands offers NIPT to all pregnant couples [2]. Prospective parents receive counseling about the option of prenatal screening, after which they must decide whether to accept or reject the screening. Besides empowering couples to make autonomous reproductive decisions, prenatal screenings can also increase anxiety and stress related to deciding whether to undergo testing, interpreting test results, concerns about the health of the baby, and the potential consequences for the baby and the family. At the time of the study, NIPT was not yet reimbursed, whereas ultrasounds were. This may explain why the uptake of NIPT screening has generally been lower in the Netherlands than in countries such as Denmark, where NIPT has been integrated into the national guidelines on prenatal screening since 2017 [3].

The current decision support for pregnant couples in the Netherlands consists of individual counseling by a trained professional, usually a midwife, along with universal information provided through a leaflet and an online questionnaire designed to prompt reflection on feelings and thoughts about prenatal screenings. For counselors, it consists of skills training, skills examination every 2 years, a counseling aid, and an e-learning tool. However, this seems insufficient for all pregnant couples to make an informed decision.

Despite the availability of counseling and informational materials, between 25% to 45% of pregnant couples in the Netherlands do not make an informed decision about prenatal screening: their decisions are value-inconsistent or based on insufficient knowledge and deliberation [4,5]. Counselors have difficulty tailoring their decision support to the needs of pregnant couples, especially those with limited health literacy skills [6-9]. More than one-third (36%) of the population in the Netherlands has limited health literacy skills, with the majority being individuals with lower levels of education or a migration background [10,11]. They have difficulty in understanding health information and in taking an active role in decision-making [12]. To overcome this gap, the Dutch Health Council—an independent advisory council that provides the Dutch government with evidence-based advice on public health and health care policy, including medical screening programs like prenatal screening—recommends more inclusive decision support [13]. Suggested strategies include (1) providing information materials that are accessible to individuals with low literacy, and (2) educating counselors to understand the challenges faced by vulnerable pregnant couples and how to address them effectively [14]. This can be achieved by using plain language, using (audio)visual rather than textual information (eg, icons, graphics, videos, and animation), and stimulating interaction via signposting (ie, framing of information in tangible actions) and tailoring (ie, presenting personally relevant information to avoid distraction) [15]. However, decision support that fits the needs of counselors and pregnant couples was not available for prenatal screening [9,16,17].

This paper describes the systematic development of the online, interactive, virtual assistant–based “Decision Aid Prenatal Screening” (in the following “decision aid”) designed to overcome this gap, and to support pregnant couples with low health literacy in making informed choices about prenatal screening.

Online, interactive, and tailored video-based decision aids have shown promise in enhancing comprehension, counseling, and informed decision-making [18-20]. A possible way to improve communication is to use video-based assistants, which offer the following advantages: they are easily accessible tools with audio-visual elements and a talking avatar who interacts with the user in a structured way. The assistant asks for user input to provide personalized information and advice, while explaining complex topics in a human-like conversation or counseling session. The key strengths of these tools are that they improve attention, comprehension, and information processing (ie, active learning): (1) the use of video promotes vividness of the information (narrative) and ease of understanding [21], (2) tailoring ensures relevance and fit to the needs of users [22], and (3) the interactive part enables scenario-based learning of the pros and cons of different screening choices [23]. Online, interactive, and tailored video-based assistants can furthermore improve provider-patient communication and informed decision-making and reduce decisional conflict [18,20,24,25]. In the paper, the online, interactive, and tailored video-based assistant will be referred to as “the decision aid.”

## Methods

### Design

This study applied a user-centered, iterative design approach guided by the Intervention Mapping (IM) framework [26,27]. User-centered design is an approach in which experiences of end users and their interaction with the intervention are central in all steps of intervention development to increase usability and effectiveness [26]. The IM framework consists of 6 steps that provide a systematic and evidence-based approach to develop, implement, and evaluate health-promoting interventions. The first 4 steps—(1) needs assessment, (2) defining change objectives, (3) selecting theoretical methods, and (4) program production—are described in this paper. Implementation (step 5) and evaluation (step 6) will be addressed in the effect evaluation (randomized controlled trial; see trial registration number ISRCTN18016226).

### Participants

#### Needs Assessment

Participants included both end users (ie, pregnant couples, including those with limited health literacy) and health care professionals (HCPs, ie, counselors). Inclusion criteria for counselors were at least 5 counselors with varying levels of experience in prenatal screening counseling, working in practices located in low socioeconomic status (SES) or disadvantaged neighborhoods. They may also include obstetric care providers, including gynecologists, with experience in prenatal screening and counseling. Inclusion criteria for end users were pregnant women from the second trimester onward (after the 20-week ultrasound) who had already made a decision regarding the NIPT, combined test, or 20-week ultrasound, and their partners. We strived to include a minimum of 18 participants, including 4 partners, 4 highly educated individuals, 1 temporary emigrant, and 9 individuals with low education (secondary vocational education level 1 or lower) or migration background (Eastern European, refugee, Turkish, or Moroccan).

#### Prototype Testing

Participants in prototype testing included individuals with limited health literacy, pregnant individuals, and partners of pregnant individuals, as well as counselors.

#### Recruitment

Participants were recruited in lower SES neighborhoods via midwifery practices, “Pharos” (the Dutch center of expertise on health inequalities), and “Stichting ABC” (a volunteer organization of and for individuals with low literacy). The selection specifically targeted people with low health literacy or limited literacy (reading, writing, and digital skills). Recruitment materials were designed in a personal style with simple words (for details, refer to [28]). Participants of the needs assessment were recruited in April and June 2020, and participants for prototype testing were recruited between February 2021 and January 2022 for various rounds of prototype testing.

#### Advisory Board

To anticipate implementation of the decision aid, an advisory board was set up at the start of the project, comprising obstetric care providers, midwives, gynecologists, counselors, researchers, and health literacy experts. Moreover, members of the working group of the program committee, Prenatal Screening of the National Institute for Public Health and the Environment (RIVM), were part of the advisory board. This working group consisted of researchers, gynecologists, and midwives who advise RIVM and the program committee on communication strategies and the promotion of professionalization in prenatal screening in the Netherlands [29]. Their role was to provide ongoing content-related feedback to ensure that the decision aid would be in line with Dutch prenatal screening standards.

### Ethical Considerations

The study and intervention research were approved by the ethical board of the Netherlands Organisation for Applied Scientific Research (Institutional Review Board 2020-035). All participants who took part in the research studies received oral and written information, including information about the possibility to opt out, and provided informed consent. Participation in the interview was confidential. This means that no personal details of participants were stored or reported, and therefore could not be traced back to participants. The information was used solely for this study. Participants received gift vouchers for participation. End users were compensated with a 25€ (US $29.41) coupon. HCPs were compensated with 65€ (US $76.47). The trial is registered at the International Standard Randomised Controlled Trial Registry with registration number ISRCTN18016226. Data were summarized and stored anonymously in a protected SharePoint vault that only the researchers could access and will be deleted after 15 years.

### Materials

Materials included interview protocols for counselors and pregnant individuals for needs assessments and testing schemes for prototype testing.

### Procedure

Development of the decision aid followed the IM steps [27] listed below.

#### Step 1

A needs assessment was conducted using semistructured interviews with end users (ie, pregnant individuals) and HCPs (ie, counselors) to identify experiences with counseling on prenatal screening and needs and wishes related to informed decision-making for prenatal screening.

Interviews with end users focused on (1) participants’ experiences with decision-making about prenatal screening, (2) the use and evaluation of decision aids and other tools such as brochures and websites, and (3) preferences for a new tool. The latter was evaluated using A/B testing (also known as split testing), a user experience method to assess performance against a specific goal (such as acceptance or functionality). A/B testing comprised providing participants with 5 different options of interactive digital tools, including diverse behavior change strategies [30]. Interviews with end users took 60 minutes. The translated interview scheme can be found in Multimedia Appendix 1.

Interviews with HCPs focused on three main topics: (1) supporting pregnant couples (with low health literacy) in prenatal screening, including challenges and a reflection on their role as counselor; (2) tools they currently use, including usability, and needs for extra tools to use before, during, and after counseling; and (3) needs and wishes for the new decision aid, including content and functionalities. Interviews with HCPs took 90 minutes. The translated interview scheme can be found in Multimedia Appendix 2.

Interviews took place online via Skype.

#### Step 2

Based on the outcomes of the needs assessment, behavior objectives were formulated for the decision aid targeting the key behavioral determinants (eg, knowledge and attitude). These were formulated into change objectives in line with the needs assessment (eg, preferences based on A/B tests), literature [30], and consensus among the project group through discussion.

#### Step 3

Theoretical methods were selected based on the determinants and translated into practical applications (functionalities within the decision aid), taking into account requirements for use. These were based on input retrieved during steps 1 and 2, literature, and consensus among the project group.

#### Step 4

A prototype was iteratively developed following a program plan. This included the script and functionalities of the decision aid. The prototype was iteratively tested with users and relevant stakeholders (ie, the advisory board and RIVM working group). The testing assessed the (1) concept of the decision aid, (2) information provided in the decision aid, and (3) functionalities. Prototype testing took place individually and was carried out by project members with expertise in health inequality. Detailed information on recruitment and testing procedures, including test methods appropriate for individuals with low health literacy, is thoroughly described elsewhere [28]. These included presenting figures and pictures to assess inferred meaning and comprehensiveness, asking participants to read-aloud and describe the information in their own words (“teach-back method”) to uncover difficult words or unclear pieces of information, giving participants assignments (such as navigating to a certain piece of information), and asking participants to provide their opinion and advice for improvement. Testing with end users took 90 minutes and was compensated with a 25€ (US $29.41) coupon. Testing took place online via Skype. All participants provided informed consent. Data was summarized and stored anonymously in a protected SharePoint vault that only the researchers can access. To anticipate implementation and use of the decision aid, an e-learning for counselors was developed, which will be briefly described.

### Data Collection

Data collection during the needs assessment entailed qualitative data through individual semistructured interviews and a focus group interview. During prototype testing (including usability testing), participants provided feedback on content clarity, usability, cultural sensitivity, and perceived usefulness collected through individual semistructured interviews. Interviews were recorded and transcribed.

### Data Processing and Analysis

Data from interviews were thematically analyzed to identify user needs and wishes, usability issues, and content gaps. Iterative refinements were made based on user input that was tested again among the end users and HCPs.

## Results

### IM Step 1: Needs Assessment

#### Participants

From the semistructured interviews with potential users (n=22), we gathered information on needs and wishes. These included (1) individual interviews with 6 counselors (mean age 39, SD 12.21 years) with an average counseling experience of 15.5 (SD 12.28) years; (2) interviews with 4 pregnant individuals with an average age of 29.75 (SD 4.11) years, who were generally higher educated (average 18.38, SD 4.27 years of education), had medical backgrounds, and represented diverse countries of origin for themselves and their partners, including in the Netherlands, Suriname, Ethiopia, Poland and Greece; and (3) a focus group interview with 12 pregnant individuals with limited health literacy due to the language barriers, who were participating in group care for Eritrean individuals (described in [28]). Interviews were conducted between July 2020 and September 2020.

#### Needs and Wishes of Pregnant Women and Counselors

The findings revealed overarching themes relating to clarity, cultural sensitivity, accessibility, expectation management, and informed decision-making.

1. False security: Both pregnant women and HCPs report that it is essential to address the so-called “false sense of security,” which may arise when screenings show no anomalies:
  *[The screenings] provide a false security, you only pick up 3 possible anomalies with it but there are up to the 1000 more possible anomalies.* [Counselor]
  The tool should thus emphasize that the screenings cannot guarantee the baby to be free of health problems beyond what was screened for.
2. Incidental findings: Pregnant women and HCPs expressed a need to explain incidental findings. These are unexpected results unrelated to the primary screening purpose:
  *Incidental findings are very difficult to grasp. What kind of incidental findings are there and what can come out of [the results] and what does that mean* [Counselor]
3. Structured information: pregnant women specifically preferred clear, concise, and well-structured information. Counselors requested the decision aid to help verify comprehension (a checklist to see if it really comes across and do they understand) or to be used during counseling.
4. Accessibility: individuals with a migrant background highlighted the importance of receiving the information in their native language, as misunderstandings caused anxiety and uncertainty about the purpose and procedure of the screenings. Many were unaware that screening is optional and “not used to receiving such screenings.” In line with end users, counselors asked for a multilingual decision aid that allows the counselor to follow along in Dutch:
*In people with moderate language skills [it is difficult to] convey the details well with proper nuances, you need to make it more manageable; A double card with the same information and the left and right in both Dutch and the another language.*
5. Cultural sensitivity: Additionally, they favored culturally sensitive information, in spoken form rather than written text.
  *Less writing but more spoken text* [End user]
6. Belief: participants with religious beliefs reported to generally declining screening due to their belief:
  “[we] *accept anything God has planned*”, a sentiment echoed by counselors who had similar experiences with non-Dutch couples.
7. Preparation: Most pregnant women favored using the decision aid digitally, especially on their mobile phone, to prepare for the counseling (together with their partner) or the birth of a child with anomalies:
  *Preparation for possible child with anomalies and you know where you stand, can arrange things and get information about it* [End user]
8. Informed decision-making: counselors emphasized the need to make pregnant couples aware that undergoing screening is a personal choice, and clarify its implications:
  *Making them aware that they have their own choice and what do they choose if they choose* [to undergo the screening]
9. Expectation management: counselors also emphasized the need to make pregnant couples aware that undergoing screening is a personal choice and clarify its implications
  *Making them aware that they have their own choice and what do they choose if they choose* [to undergo the screening]
  They reported the observation of couples misunderstanding the purpose of screenings, such as assuming that ultrasounds are to determine the baby’s sex:
*Sometimes people assume that [at the ultrasound] they just get to know the sex. Then they point out that there may also be findings such as a heart defect so that people are prepared and aware of it.*
  Expectation management may prepare pregnant couples for potential bad news relating to fetal anomalies uncovered during the ultrasounds.

### IM Step 2: Change Objectives

The intervention’s goal was to promote informed decision-making among pregnant couples prior to the decision regarding prenatal screening [31,32]. We formulated subgoals to be addressed by means of the decision aid, based on the needs assessment and literature (see Table 1 for an overview of the different goals). Definite change objectives were derived by consensus forming among the project group, in line with relevant literature. Project group members included researchers, a professor of midwifery, a former midwife and teacher in midwifery, and experts on health inequalities.

**Table 1:** Definition of program objectives and selection of change methods for the decision aid for pregnant couples.

Key objectives	Key determinants	Change objectives	Method: practical application
Actively review information regarding prenatal screening, health conditions, and anomalies	Knowledge	Are able to explain what health conditions and anomalies entail and what these mean for the life of the baby itself and the family Are able to explain what the prenatal screenings entail and how they are carried out Recognize that opting for or against prenatal screening is a personal choice that they are involved in	Consciousness-raising [33,34]: Factual information concerning (1) the screening (aim, procedure, and implications), (2) diseases and anomalies (probabilities, implications for life of baby and family, certainty and false sense of security of test results), and (3) freedom of choice that fully lies with the pregnant couples, including potential consequences of opting and not opting for the screenings Chunking [35]: Providing small pieces of information, aggregated in logical groups (eg, “What is Down Syndrome?”, “How is it for your child?” “How is it for your family?”; see Multimedia Appendix 3) Tailoring [36,37]: Tailoring by (1) allowing the user to navigate to the most relevant information, split in logical chunks by means of information routing, and (2) providing users with personalized summaries and insights in the Modules “Help me decide” and “Further questions” Teach-back method [38,39]: Chunk “Did I explain it well?” to repeat the most essential information (without users experiencing text anxiety)
Weigh the pros and cons of screening and possible results	Attitude	Are able to explain what the benefits and disadvantages of undergoing prenatal screening are for themselves Are able to elaborate on the benefits and disadvantages of potential pregnancy termination in their personal situation	Value clarification [40,41]: Unbiased decisional balance sheet in module “Help me Decide”: users are prompted to weigh pros and cons to consider relevant aspects within their decision, for example, “I want to have the option to terminate the pregnancy if my baby has a serious anomaly” or “I want to be able to prepare for life with a child with a serious anomaly” Narrative information and role model stories [23]: Provision of various audio-visual scenarios depicting a variety of situations, perspectives, and respective implications
Discuss questions with significant others (eg, partner, midwife) in case information is unclear, or there are questions with regard to the personal situation, prior to the decision	Self-efficacy	Express confidence in discussing (remaining) questions with significant others (eg, partner, midwife)	Facilitation [42,43]: Facilitation through (1) module “Remaining questions”: stimulating users to think about remaining questions they might have, such as “Why does testing not guarantee that my baby is healthy?” or “What can I do if my partner does not agree with my decision?” and to bring these questions to their counselor for further discussion. (2) Module “Help me decide”: preparing pregnant couples to discuss important values, thoughts, and concerns regarding prenatal screening with each other, and the counselor
Make a decision for or against the different screenings in agreement with their partner	Self-efficacy	Express confidence in making a well-informed choice based on knowledge and attitude about using the screening tests, in agreement with each other	Value clarification [40,41,44]: Unbiased decisional balance sheet in module “Help me decide”; see above

### IM Step 3: Program Design

#### Theoretical Methods and Practical Applications

In line with the outcomes of the needs assessment, the set of objectives was translated into a content script and functionalities. We aimed for an online, interactive, and tailored video-based decision aid for pregnant couples that they can use to prepare for the consultation. The content of the decision aid was based on the behavioral determinants (defined in Table 1) and a selection of appropriate methods fitting these targeted determinants. Appropriate methods were chosen based on the literature and consensus forming among the project group. A first draft of the script of the decision aid and functionalities was written by the project group members. This included information on the prenatal screening tests, the fetal anomalies, and the consequences for the baby and the family. The script also included relevant and frequent considerations to support the user in decision-making and frequently asked questions. The script was iteratively reviewed by the project group and the advisory board, including the RIVM working group, and tested with end users. Adaptations were made based on feedback from the professionals, in line with input from user testing (as described in step 4). The script was then implemented as a spoken version to be tested in the prototype.

We followed International Patient Decision Aid Standards criteria for decision aids [42], (1) ensuring information provision of all options, (2) presenting information about outcomes in an unbiased and understandable way, (3) providing methods for clarifying patient values, and (4) including guidance for deliberation and communication (Table 1). Below, we discuss several examples of methods and strategies applied to address the change objectives derived from steps 1 and 2.

#### Examples for Methods and Strategies Used

As an example, to target knowledge, we used consciousness-raising [33,34] about the (1) screenings including their aim, procedure, and implications (including false sense of security); (2) the fetal anomalies, what these entail, and what the life of a child and family with the former may look like; and (3) freedom of choice that fully lies with the pregnant couple, encompassing potential consequences when opting in or out of the screening. This incorporated factual knowledge concerning, among others, probabilities of anomalies, certainty of test results, and difficulties that children or (grown up) individuals and families with the anomalies may face.

Attitude formation was promoted by stimulating value clarification, for which we used an unbiased decisional balance sheet (eg, [40,41,44]) in the “Help me decide” module. This neutral method for weighing pros and cons of (not) acting on a certain kind of choice stimulates decision-making readiness and lowers decisional ambivalence or conflict. Users are therefore required to think of which aspects of the choice regarding prenatal screening matter to them (eg, knowing as soon as possible if their baby has an anomaly). Value clarification was further supported using information regarding screening procedures and potential outcomes (ie, how does screening work and what is it like for the child and family).

Self-efficacy to communicate with a counselor or a partner was enhanced by stimulating users to think about remaining questions they might have in the “Further questions” module, such as “Why does testing not guarantee that my baby is healthy?” or “What can I do if my partner does not agree with my decision?” and pose these questions to their counselor or partner. Moreover, the “Help me decide” module encourages users to consider relevant aspects within their decision, for example, “I want to have the option to terminate the pregnancy if my baby has a serious anomaly,” or “I want to be able to prepare for life with a child with a serious anomaly” to prepare them for a conversation with their partner and counselor. Additionally, self-efficacy was tackled by narratives, which can increase self-efficacy and engagement [45].

**Figure 1:**
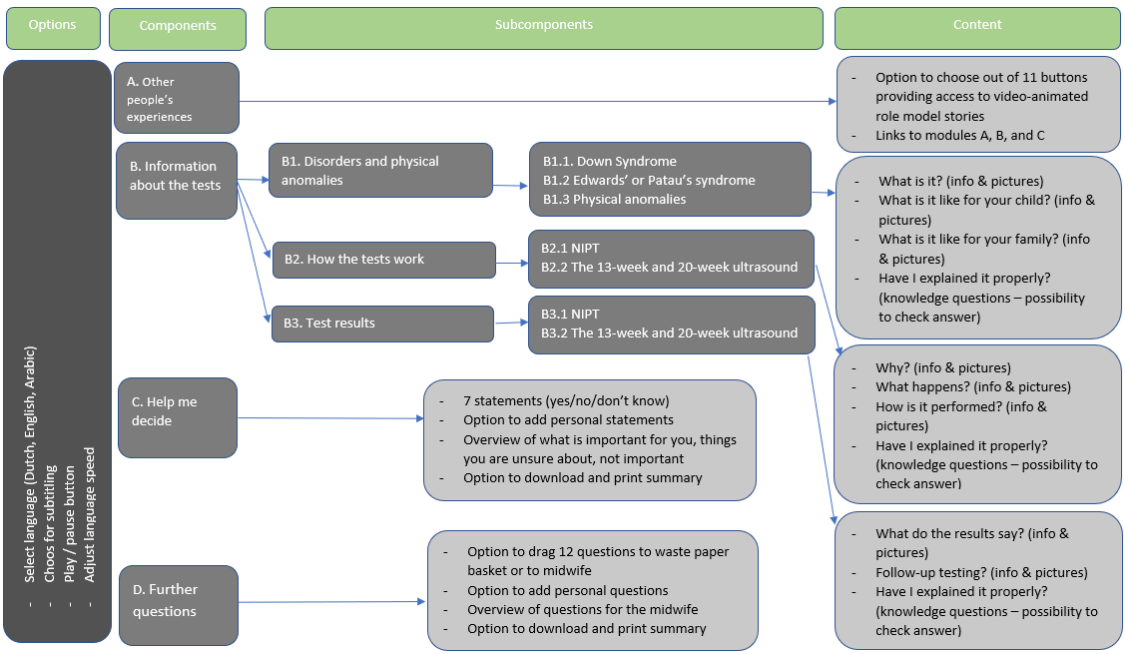
Blueprint of the decision aid for prenatal screening. NIPT: noninvasive prenatal testing.

To increase the overall accessibility of the decision aid, it was important to limit the amount of information, offer the content in various languages, and present it audiovisually [21]. We therefore opted for an avatar to verbally explain the use of the decision aid and to guide users through the information, using additional visual aids such as pictures (eg, of a vitality ultrasound) and video scenarios (eg, of role model experiences). Using avatars in digital interventions offers various benefits such as increased information recall and improved learning compared to static content [25,46,47]. Besides, avatars have previously been shown to increase behavior change and literacy [25,48-50] alongside making the user experience more engaging and enjoyable [25,49,51,52]. Moreover, avatars have previously been shown to promote a feeling of connectedness and support (eg, by providing more personalized feedback) [53-57] and have the potential for increasing the effectiveness of an intervention [25,48,49,58]. To enhance information processing and retrieval, and to make the decision aid quick to go through, as requested, we applied chunking throughout the decision aid by grouping information into logical chunks of information [35]. The “Help me decide” module and “Further questions” module make use of interaction to stimulate scenario-based and active learning [23]. The use of narratives by means of personal stories in the module “Experiences of others” was meant to promote greater engagement with information, as these may be especially useful for people with limited literacy skills. A complete overview of the final decision aid sections can be found in Figure 1.

### IM Step 4: Prototype Testing and Program Production

The decision aid was created using user-centered design and iterative testing with ongoing involvement of potential end users, counselors, and relevant stakeholders for implementation. In this step, we will describe the final decision aid (see Multimedia Appendix 3 for screenshots of the decision aid). As part of step 4, we evaluated 3 prototype versions in different stages of development with potential end users (ie, pregnant couples) and intermediate users (ie, counselors).

#### Prototype Testing

Three prototypes were iteratively tested between March 2021 and July 2022 among individuals with limited health literacy skills (n=4), (pregnant) women (n=6), and fathers (n=2) from lower SES neighborhoods, and counselors (n=4). Additionally, the advisory board and the working group provided ongoing feedback. Table 2 provides an overview of prototype testing rounds, including the aim, sample, methods used, results, and adaptations.

**Table 2:** Overview of iterative development phases of the decision aid, including feedback provided by various target users and adaptations.

Round	Aim	Sample	Methods used	Results	Adaptations
1	Testing the goal, ease of use, sound clips, and overall impression. Thereby assessing: homepage, main menu, information provided by the avatar about the screening pictures, and reading aloud functionality	3 individuals with former limited literacy, 1 pregnant woman	Teach-back method, tasks and recommendations, open questions	Goal of the decision aid is unclear^a^ Lack of essential navigation features Sound fragments are too long Pictures are distracting and do not match the text well^a^ Generally, no difficult words^a^ Request for multilanguage decision aid^a^ (Role of) Avatar unclear^a^	Introduction of the decision aid, including the goal and functionalities Play and pause button added Pictures were changed or removed Decision aid translated into English and Arabic Avatar introduces herself and the decision aid
2	Testing the information module in written format, the name of the decision aid, and the knowledge test	3 pregnant women, one mother with former limited literacy	Teach-back method, recommendations, read-aloud, open questions	Information too long^a^ Too negatively framed (including terms “illness” and “handicap”)^a^ Change words like “tests” to “control” or “examination”^a^ Quiz questions are valuable, but can also put pressure on users Attention for emotion regulation and how to discuss this^a^	Information fragments shortened More neutral framing of information, removal of diminutives Use of teach-back questions per information fragment
3	Home screen, ‘Help me decide’ module, ‘Further questions’ module	4 midwives or counselors, 4 potential users (1 pregnant woman, 1 mother, 2 fathers)	Teach-back method, recommendations, read-aloud, open questions, think-aloud, questions regarding acceptance and future use	Expectation of added value of the decision aid for future use in practice^b^ Mention and involve the partner explicitly, earlier and more often in the decision aid^a,b^ Use more visual support^a^ Help me decide is appreciated, but response options require reframing^a^ Name of the decision aid is unclear^a^ Missing experiences of others^a^	Addition of consideration for the nonpregnant partner and naming the partner more explicitly throughout the decision aid Response options in the “Help me decide” module reframed Change of name Addition of others’ experiences

^a^Input provided by participants with (former) low literacy.

^b^Input provided by counselors.

#### Most Important Adaptations

To improve clarity, cultural sensitivity, emotional relevance, and accessibility, several adaptations were made to the decision aid, based on user feedback:

1. Title revision: the name of the decision aid was revised from “Do you want to get free tests to see if your baby is healthy?” to “Decision Aid prenatal screening: support in choosing whether to test for conditions or anomalies in your unborn baby” to better reflect its purpose.
2. Avatar redesign: the design was adapted to resemble an end user's recognizable counselor, wearing a clinical white uniform.
  *Put on midwife’s clothes. White uniform* [End user]
  This conflicted with advisory board feedback, which favored a more realistic and approachable appearance. To clarify its role, the avatar now introduces herself and explains the tool’s purpose and navigation options (eg, language and speech settings).
3. Visual and textual adjustments: Images depicting advanced pregnancy stages were replaced to better reflect the early phase of prenatal screening, in order to improve recognition.
  *We are at the beginning of the examinations. She has such a big belly. It looks like she's going into labor, that's not right* [End user]
  Text fragments were shortened for clarity.
4. Neutral language: In the “Help me decide” module, complex or emotionally charged statements were simplified to neutral, single-sentence considerations (eg, “A life with a child with Down syndrome would be too heavy for me, I therefore would like to undergo screening” to “Living with a child with a serious anomaly seems too hard for me”). Earlier versions were perceived as overly negative or steering by end users:
  *It is a sensitive topic and therefore it is important not to make judgments about the choices and severity of the anomalies* [End user]
  Revised content presents information objectively while acknowledging potential challenges. Diminutives (eg, “kiddo”) were replaced with more neutral terms (eg, “child”).
5. Inclusion of personal narratives: As requested by end users (“*I miss experiences, the feelings* […] [End user]”), personal experiences of others deciding for and against prenatal screening to help them reflect and put their emotions into perspective, were added as an extra module: Experiences of others. This module features animated scenarios based on real-life narratives that illustrate diverse perspectives (including perceived barriers, positive, and negative experiences) and decisions around the screenings.
6. Partner involvement: Both counselors and male participants emphasized the importance of involving the nonpregnant partner more explicitly, earlier, and more often in the decision aid.
  *Involving dads is very important to me* [Male end user]
  The decision aid now includes references to partners in tailored content, considerations, and remaining questions that reflect shared decision-making (eg, scenario “In my mind, I was already a father – until my girlfriend called” or consideration “What can I do if my partner wants something different than me*?*”).
7. Replacement of knowledge test by a teach-back module: To prevent test anxiety, the original Test your knowledge module was removed to prevent test anxiety. It was replaced with the teach-back prompts in each section, allowing users to confirm understanding without feeling tested.

### The Final Decision Aid

### Overview

The decision aid prenatal screening is a tailored, online, and interactive virtual assistant–based tool [59] featuring virtual assistant Anna. It can be used before, during, or after counseling and contains 48 minutes and 58 seconds of optional content that users can engage with depending on their needs and preferences. The decision aid comprises four modules: (1) Other people’s experiences, (2) Information about the tests, (3) Help me decide, and (4) Further questions (refer to Figure 1 for a blueprint of the decision aid content).

### Module 1

“Other people’s experiences” offers the user 11 different audiovisual scenarios, each lasting between 9 and 45 seconds. These depict other parents’ decisions over prenatal screening, including experiences relating to child loss, reasons for undergoing or declining screening, and choices about continuing or terminating the pregnancy. Characters and stories reflect (cultural) diversity.

### Module 2

“Information about the tests” is organized into three sections: (1) “Disorders and physical anomalies,” (2) “How the tests work,” and (3) “Test results.” The first section explains Down syndrome, Edwards’, or Patau syndrome, and physical anomalies, with details about the disorder or anomaly, and its impact on the child and family. These information chunks are combined with the teach-back question “Have I explained everything properly?” where Anna asks questions and provides answers to reinforce understanding. The second section covers the NIPT and ultrasounds, explaining what they are, why they are performed, and how they are conducted. It also includes “incidental finding” (NIPT) and “What happens at the ultrasounds?” (ultrasounds), concluding with the teach-back question. The third section explains test results (NIPT and ultrasounds), including what the results mean and potential follow-up steps, concluding with the teach-back question. All content is based on RIVM brochures and counselor experiences.

### Module 3

In the “Help me decide” module, users are guided through 7 different reasons that may influence their decision, such as accepting a child with an anomaly, preparing for life with a child with a serious anomaly, or feeling unable to cope with such a situation. Users are informed they can choose to participate in some, all, or none of the screenings. They can then select which reasons are important for their decision, and add their own additional reasons. Users are then presented with a summary that can be printed and brought to their counseling session. The module allows for cultural sensitivity, accommodating considerations fitting various cultures (including accepting the child as it is vs. choosing to abort), and the option to add one’s additional reasons.

### Module 4

“Further questions” supports users in identifying remaining questions that they would like to discuss with their counselor. Users are presented with 12 questions, which they can discard or save by dragging them to the trash can or avatar. The avatar reads each question aloud when it appears on the screen. Again, relevant questions are summarized. Examples include “What can I do if something is wrong with my baby?” or “Why does testing not guarantee that my baby is healthy?”

### Accessibility Features

To support users with limited health literacy, the decision aid includes features such as audio narration by the avatar and adjustable speed of speech. To further allow for cultural sensitivity, it is available in Dutch, English, and Arabic, with optional Dutch subtitles to assist counselors when couples use the aid together during a counseling session, in another spoken language.

### Implementation Strategy: e-Learning for Counselors

To anticipate the implementation of the decision aid and to conform with the needs and wishes of the counselors, an e-learning tool for counselors was iteratively developed and tested alongside the decision aid. The e-learning tool supports counselors in recognizing signs of potential health illiteracy and strategies on how to counsel individuals with low health literacy. Furthermore, the e-learning tool informs counselors how they can deploy the decision aid as educational material to be used before, during, or after the counseling. An overview of modules and content of the e-learning tool can be found in Multimedia Appendix 4.

## Discussion

### Principal Findings

In line with our objective to support informed decision-making among pregnant couples with low health literacy, we systematically developed and iteratively refined a tailored, video-based decision aid. Our findings demonstrate that systematically involving end-users and counselors throughout the development process contributed to building an acceptable and accessible tool that fits a diversity of needs. These include demand for clear, culturally sensitive, and accessible information, which is delivered digitally and in multiple languages to support informed, realistic decision-making about prenatal screening. The final decision aid was designed to be used during counseling, as preparation before counseling, or as a means for reflection afterwards. It includes factual information, experiences of others, support in decision-making, and formulating remaining questions. The decision aid stimulates users to think about personally relevant questions to promote active participation in the counseling, facilitate shared decision-making, and ensure an informed choice is made. Prototype testing indicated high acceptability and usability among both pregnant couples and counselors, suggesting the decision aid’s potential to fill a critical gap in current prenatal screening support. By describing the systematic design, we aimed to contribute to calls for improving the quality of reporting of the content and design rationale of interventions, to ensure reliable implementation and replication [60,61].

### Strengths

To begin with, the decision aid was developed to support pregnant couples with low literacy in making an informed decision about prenatal screening. Easily accessible and understandable information for this often underserved group is especially needed to overcome health inequalities [62]. Including individuals with limited health literacy skills in both developing and testing the decision aid enabled us to fully accommodate the design and functionalities to their needs, and to improve accessibility in general. The iterative, participatory approach resulted in the selection and application of strategies to address health literacy challenges, such as features such as audio-visual content, the teach-back strategy, and chunking. The needs assessment highlighted the importance of explicitly including partners and stories of others. First, the inclusion of partners could foster shared decision-making. Moreover, the desire for stories of others aligns with findings elsewhere, where narratives not only improve knowledge and confidence but also improve the perceived relevance and supportiveness of health information [63]. By directly involving end-users in prototype testing, we were able to identify and address barriers related to language, cultural beliefs, and digital skills. The user-centered approach and design principles allowed for meeting the needs and wishes of both pregnant couples and counselors. Iterative development and testing, moreover, contributed to increased user acceptance and overall usability, underscoring the importance of participatory design [64].

The Intervention Mapping approach ensured the intervention was grounded in behavioral theory and evidence by means of a structured, systematic approach, which increases the likelihood of addressing the most important determinants of informed decision-making [27]. Moreover, IM facilitated the integration of the perspectives of end users and counselors in every stage. Also, IM made the development process transparent and replicable. Finally, the IM approach encourages the consideration of implementation factors, such as the counselor's e-learning. The involvement of the advisory board, counselors, and the RIVM working group enabled us to anticipate future implementation of the decision aid in practice. Whether the decision aid also contributes to more effective informed decision-making remains to be seen.

### Limitations

A first limitation of the decision aid and its design approach includes the difficulty of fitting the sometimes opposing needs and wishes of both the users (ie, pregnant couples), counselors, and advisory board, such as relating to the visual appearance of the avatar. In line with the decision aid’s aim to support pregnant couples, their wish for the avatar to be better recognizable as a counselor was followed, which means that the avatar got a rather clinical look. Second, due to the development taking place during the COVID-19 pandemic, all research activities, including the needs assessment with low-literacy individuals, had to take place online, which was expressed to be a challenge by this group. To mitigate its impact, we invited a facilitator and translator who knew the participants, if necessary, to make the participants feel more comfortable, and took extra time to get to know each other, explaining the research and testing the decision aid. Thirdly, another limitation is the fact that the functionality to drag and drop questions to the midwife or wastepaper basket in the ‘Further questions’ module was not usable on iPhones, due to technical issues that could not be repaired. To facilitate users to still use this module if desired, they were referred to the browser version of the decision aid: “Unfortunately, this section does not work on iPhone. You can use this section on your desktop.”

### Practical Implications and Future Directions

The decision aid can be integrated into routine counseling, potentially improving informed choice rates and reducing decisional conflict. Its design aligns with national recommendations for inclusive, low-literacy-friendly health communication and supports the broader goal of reducing health inequalities. The methodology—combining Intervention Mapping with user-centered design—serves as a model for developing other digital health tools targeting vulnerable groups. Early consideration of implementation factors increases the likelihood of successful adoption and sustained use in practice [64].

Regarding future research, we suggest that the final decision aid should be pretested as a whole instead of its parts and functionalities, and that it will be tested in practice. In theory, the decision aid aligns with the user group as tested during the iterations; however, it should also be tested by both pregnant couples and their counselor in a real-life setting, instead of an online setting, that is, before, during, and after the counseling. It would further be interesting to assess which of the modules are necessary for users to make an informed decision as the ‘most essential elements’ of the decision aid. Lastly, the effect of the decision aid on behavioral determinants such as knowledge, attitude, and informed decision-making should be researched. The effects of this trial will be described in a following article.

### Conclusion

This study presented the systematic, user-centered development of a decision aid designed to support pregnant couples with low health literacy in making well-informed choices about prenatal screening. By integrating evidence-based techniques and tailoring the tool to the needs of underserved populations, the decision aid could contribute to reducing health inequalities and promoting shared decision-making in prenatal care. The development process, grounded in Intervention Mapping, demonstrates how structured, iterative design can enhance usability, accessibility, and stakeholder engagement. Ultimately, this work contributes to the broader goal of equitable health care by offering a replicable model for designing inclusive decision support tools that align with the needs of diverse populations.

## Appendix

Multimedia Appendix 1 Interview scheme needs-assessment pregnant women.

Multimedia Appendix 2 Interview scheme needs-assessment HCPs.

Multimedia Appendix 3 Screenshots of the decision aid.

Multimedia Appendix 4 Table 3. Overview of modules and learning goals of the e-Learning.

## Abbreviations

HCP: health care professional
IM: Intervention Mapping
NIPT: noninvasive prenatal testing
RIVM: National Institute for Public Health and the Environment
SES: socioeconomic status
ZonMw: Care Research Netherlands and Medical Sciences

## Data Availability

The datasets generated or analyzed during this study are not publicly available due to privacy protection, but are available from the corresponding author on reasonable request.
