# Supplementary material for: Development of a Tailored Online Video-Based Assistant to Support Prenatal Screening Decisions in Couples With Limited Health Literacy: User-Centered Design Approach
Source: JMIR Form Res. 2026 Mar 27;10:e75391. doi: 10.2196/75391 (PMC13069372; doi:10.2196/75391)
Supplement: Multimedia Appendix 1 [file formative_v10i1e75391_app1.docx]

## Multimedia Appendix 1.

Interview scheme needs-assessment pregnant women

### Interview scheme for pregnant women or their partners on Prenatal Screening (PS) (Screening for Down, Edwards, and Patau syndromes, and the 20-week ultrasound): Experiences with current decision-making, available tools, and needs for the online video intervention

#### Interview Goals

- Identify experiences in making a choice about prenatal screening among pregnant women who have difficulty understanding information about PS.
  - What are the experiences and challenges regarding decision-making, interpretation of risks, and follow-up actions related to PS?
- Identify experiences with (any) tools used in decision-making about PS.
  - Which tools do vulnerable pregnant women use? And what are the experiences and challenges with using them?
- Identify needs and wishes for the online, interactive, tailored video intervention for pregnant women (and their partners).
  - What are the needs and wishes for the intervention in terms of content, functionality, and format?
  - What are the requirements the intervention must meet in terms of content, functionalities, and format?

#### Target Group

#### Pregnant women from the second trimester onward (after the 20-week ultrasound) who have already made a decision regarding the NIPT/Combined Test/20-week ultrasound, and their partners.

#### Minimum of 18 participants, including 4 partners, 4 highly educated individuals, 1 temporary emigrant, and 9 individuals with low education (MBO level 1 or lower) and/or migration background (Eastern European/refugee/Turkish/Moroccan).

#### Method

Individual interviews conducted at the participant’s home or online. *[Eventually carried out online due to COVID-19].*

#### Duration

Approximately 60 minutes

#### Materials

- Current tools for midwives and pregnant women: brochure on prenatal screening (https://www.pns.nl/down-edwards-patau-en-seo/professionals/documenten)
- Example tools to illustrate wishes/needs/requirements regarding possible functionalities
- Informed Consent: oral or via form (see the form for essential components)
- Bol.com gift card
- If applicable: travel reimbursement form
- Laptop/tablet (to show examples)
- Recording equipment (voice recorder)
- For oral consent: record separately and store apart from the interview.
- For written consent: send in advance and request signed return before the interview.

### Interview Structure

Welcome & Introduction (10 min)

Part 1: Experience of Pregnant Women with PS: Decision-making, interpretation of risks and actions (25 min)

Part 2: Needs for the intervention (20 min)

Closing (5 min)

#### Welcome + introduction

- Welcome participant
- Introduce yourself (interviewer)
- Explain interview purpose: “TNO, Pharos, and Maastricht Midwifery Academy are developing a program that guides pregnant women in making a choice about screening for fetal anomalies in their unborn child (also known as prenatal screening): screening for Down syndrome, Edwards syndrome, and Patau syndrome (around 12 weeks of pregnancy), and the 20-week ultrasound.”
- Overview of interview topics: During your pregnancy, you will be offered various screenings. You can have tests done to determine the likelihood of your child having Down syndrome, Edwards syndrome, or Patau syndrome. The 20-week ultrasound can be used to check whether your child has any physical anomalies. We are interested in hearing about your experiences with making decisions about these tests/screenings. The interview consists of two parts: your experiences with making decisions about the screenings, and your needs for the new program.
- Explain recording and privacy: The interview takes approximately 60 minutes. It will be recorded and data will be anonymized. This means that your name will not be reported anywhere in the report. The recording will be deleted after summarizing all information. Participation is voluntary, and you may stop at any time. For participation start we require your informed consent. If you have any questions you may pose them anytime.
- Confirm consent (if online, already received).

#### Background Questions

Before we start I would like to ask some background questions.

-What is your age? What is your partner’s age?

-What is your country of birth? What is the country of birth of your partner?

-What is your highest level of education? How many years of education have you completed? Include the years of elementary school.

-What is your partner’s highest level of education? How many years of education has your partner completed? Include the years of elementary school.

#### Part 1: Experience of Pregnant Women with PS: Decision-making, interpretation of risks and actions (25 min)

**Introduction questions**

• How is your pregnancy going?

• How many weeks pregnant are you now?

• Is this your first child?

**Knowledge about prenatal screening**

- Have you ever heard of prenatal screening?

*Let me explain.*

*During pregnancy, women can have their unborn child tested for fetal anomalies. There are various tests you can do. Such as the NIPT or Combination Test. The midwife will then assess the likelihood of the baby having physical anomalies. This is the test for Down syndrome, Edward syndrome, and Patau syndrome. Another test is the 20-week ultrasound. The midwife uses an ultrasound to check if everything is okay with your baby. For example, they can see if the baby has spina bifida (open back) or anencephaly (open skull).*

*The NIPT or combination test and 20-week ultrasound are not mandatory. Pregnant women can decide for themselves whether or not they want to have the test done.*

*Ideally, the midwife will have already explained all of this to you and you will have already made a decision about whether you want to do it.*

**NIPT/Combination test**

Do you remember talking to the midwife about the NIPT or combination test?

[follow-up questions]

- Did you understand what she told you about it?
- What did you understand and what didn't you understand?
- What did you like about the conversation? What did you like less?
- Was that during a specially scheduled conversation? Or during a normal appointment?

• Did the midwife talk about... ?

- That you can choose whether you want it?
- What you have to do for it? (blood test)
- Adverse findings? If so, do you know what that is?
- Costs?

Did you have the NIPT or combination test (screening for Down, Edwards, and Patau syndromes)?

[follow up questions]

- Why did you/didn't you have it done?

[consider: cost, no intention to act on the results, religious beliefs, social environment]

• Can you list some positive aspects of the screening?

• Can you list some negative aspects of the screening?

• Did you find it difficult to make the decision? Why/why not?

**20 week ultrasound**

Do you remember talking to the midwife about the 20-week ultrasound?

[follow-up questions]

- Did you understand what she told you about it?
- What did you understand and what didn't you understand?
- What did you like about the conversation? What did you like less?

• Did you have the 20-week ultrasound?

[ask follow-up questions]

- Why did you/didn't you have it?

[consider: costs, no action to be taken based on the results, religious beliefs, social environment]

• Did the midwife discuss... ?

- That you can choose whether you want it?
- What you need to do to have it? (ultrasound)
- Follow-up examination?
- Costs?

• Can you list some good points about the screening?

• Can you list some negative points about the screening?

• Did you find it difficult to make the choice? Why/why not?

**Information sources**

- Did you have enough information to make your decision?

[follow-up question] Why/why not? What else would you have liked to know?

- How did you obtain that information? [follow-up question: why and how was that (understandable)]

o Midwife

o Partner

o Brochure

o Family/acquaintances/friends

o Internet

- Do you remember which website(s) you visited? (e.g., www.pns.nl or www.onderzoekvanmijnongeborenkind.nl).

o Other….

*[Show the PED & 20-week ultrasound brochure + explain that you will receive this before making your decision (https://www.pns.nl/down-edwards-patau-en-seo/professionals/documenten).]*

- Did you receive and read the brochure about the screening?

- If participant read it:

- It contains complex information, and many people find it difficult to understand. Can you understand what it says? What do you understand and what don't you understand?
- What did you like and dislike about the brochure?

- If participant hasn't read it or can't remember: ask the participant to go through the brochure.

- What is your first impression? What strikes you?
- What do you like and dislike about the brochure?
- It contains some rather difficult information, which many people find hard to understand. If the brochure has been received/viewed previously: Can you understand what is written here? What do you understand and what don't you understand?

Have you discussed the screening with others, such as your partner, family, friends, doctor, or other healthcare providers?

- Who did you talk to?

[Follow-up questions: Why? What did you like about this? What did you like less?]

- Did they influence your decision? (how?)
- Did you do anything else to help you make your decision (e.g., fill out an online decision aid/questionnaire)? Why/why not? And what? And what did you think of this?

What ultimately helped you most in making your decision? And why?

o Conversation with midwife

o Conversation with partner

o Conversation with others

o Reading information in brochures

o Reading information online

o Watching a film online

o Completing an online questionnaire (decision aid)

#### Part 2: Needs for the intervention (20 min)

We are creating new information about prenatal screening for pregnant women and their partners via the internet. This information can be used by you and your partner to make a decision about prenatal screening. We would like to know what you consider important in this information.

We will now show you a number of examples and ask you for your first impression. These examples are not about prenatal screening. Therefore, try not to focus on the content, but on the way in which you are guided through the information and how it is presented. We would like to know what you think about this and whether it could also be suitable for prenatal screening. There are no right or wrong answers; you can say whatever comes to mind.

##### Oudercoach: https://oudercoach.guidingtube.com/

[Show, let them experience the home page and 1 video (2 questions + feedback)].

-What is your first impression?

-What appeals to you and why?

-What doesn’t appeal to you and why?

Suppose we create this for prenatal screening so that you and your partner can use it to make a decision.

- Would you use it? + Why/why not?

- What do you like and dislike about it?

Which smiley would you give this example + why?
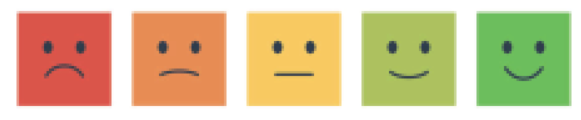


##### 22-weeks vaccination: <https://tno-kinkhoest.aanzee.online/>

##### Prepare conversation

[View and let them use functionality “prepare conversation” section: [*https://tno-kinkhoest.aanzee.online/in-gesprek*](https://tno-kinkhoest.aanzee.online/in-gesprek)*]*

-What is your first impression?

-What appeals to you and why?

-What doesn’t appeal to you and why?

Suppose we create this for you and your partner so that you can prepare for the conversation with your midwife/gynecologist (or with others who are important to you) about prenatal screening:

- Would you use it? + Why/why not?

- What do you like and dislike about it?

Which smiley would you give this example + why?
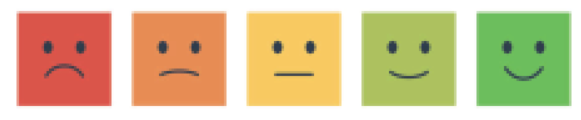


##### Advantages & disadvantages

[View and let them use functionality “advantages and disadvantages” <https://tno-kinkhoest.aanzee.online/voor-en-nadelen>]

-What is your first impression?

-What appeals to you and why?

-What doesn’t appeal to you and why?

Suppose we create this for you and your partner to weigh the pros and cons of prenatal screening so that you can make the right choice for you:

- Would you use this + why/why not?

- What do you like and dislike?

Which smiley would you give this example + why?
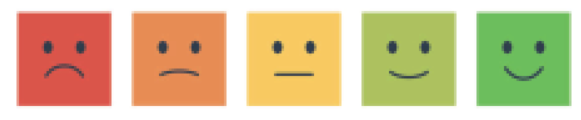


##### Knowledge Test

[View and let them use functionality “Knowledge test” section: [*https://tno-kinkhoest.aanzee.online/test-je-kennis*](https://tno-kinkhoest.aanzee.online/test-je-kennis)]

-What is your first impression?

-What appeals to you and why?

-What doesn’t appeal to you and why?

Suppose we create a knowledge test for you and your partner about the screenings so that you can check whether you have understood the information about the screenings correctly.

- Would you use this + why/why not?

- What do you like and dislike?

Which smiley would you give this example + why?
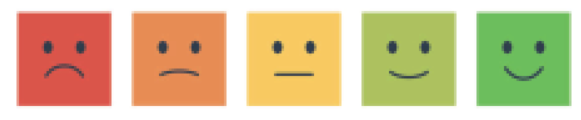


##### Talent in Huis

[Show and let them go through beginning to getting to know the first person: <https://www.talentinhuis.nl/>]

-What is your first impression?

-What appeals to you and why?

-What doesn’t appeal to you and why?

Suppose we create this for you and your partner, so that you and your partner can gather more information about prenatal screening. You will then talk to a midwife, such as at Talent in Huis, and you can gather information, ask questions, or weigh up the pros and cons together so that you can make a choice:

- Would you use this + why/why not?

- What do you like and dislike?

Which smiley would you give this example + why?
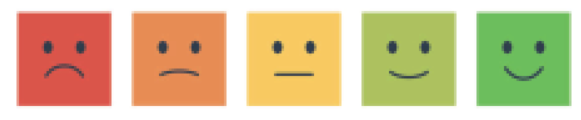


#### General Questions

You saw some examples of (digital) tools.

-Which example appealed most to you and why?

-Which appealed least and why?

-Which format do you prefer? Text, images, audio, more video? And why?

-Would you do this together with your partner? Why/why not?

-Would you do this together with your midwife or at home (or both options)? Why?

-When would you like to use the information (as preparation, during a consultation with your midwife, afterwards)?

-How would you like to access it, for example via telephone, computer, or laptop? And why?

-How much time would you like to spend on it? Why?

-What topics would you like to receive information about? Why? Think about things you encounter. What challenges/problems should it solve for you that you may have encountered?

-Do you have any questions or tips that have not been addressed?

#### Closing (5 min)

-Thank participant

-Ask if they have remaining questions

-provide gift card (or collect details to send if online)

-For questions or comments, contact: [researcher information]
