# Supplementary material for: Development of a Tailored Online Video-Based Assistant to Support Prenatal Screening Decisions in Couples With Limited Health Literacy: User-Centered Design Approach
Source: JMIR Form Res. 2026 Mar 27;10:e75391. doi: 10.2196/75391 (PMC13069372; doi:10.2196/75391)
Supplement: Multimedia Appendix 2 [file formative_v10i1e75391_app2.docx]

## Multimedia Appendix 2.

Interview scheme needs-assessment HCPs

### Interview scheme for Counselors on Prenatal Screening (Screening for Down, Edwards, and Patau syndromes, and the 20-week ultrasound): Experiences with current decision-making, available tools, and needs for the online video intervention

#### Interview Goals

- Identify how midwives recognize pregnant women who struggle to understand information about prenatal screening.
- Explore midwives’ experiences in supporting these women with decision-making.
  - Understand challenges related to decision-making, interpreting risks, and follow-up actions.
- Gather experiences with tools used to support decision-making.
  - Learn which tools midwives use (for themselves and for vulnerable pregnant women).
  - Understand experiences and challenges with using these tools.
- Identify needs and preferences for an online, interactive, tailored video intervention to better support these women.
  - Determine desired content, features, and format of the intervention.
    - Define requirements for accessibility and usability.

#### Target Group

At least five midwives/counselors with varying levels of experience in prenatal screening counseling, working in practices located in low socioeconomic status (SES) or disadvantaged neighborhoods. May also include obstetric care providers, including gynecologists, with experience in prenatal screening counseling.

#### Method

Individual interviews conducted at the midwife’s or gynecologist’s home or practice. *[Eventually carried out online due to COVID-19].*

#### Duration

Approximately 90 minutes

#### Materials

- Example tools to illustrate desired features and requirements
- Informed consent (oral or written)
- Gift card
- Travel reimbursement form (if applicable)
- Laptop or tablet to show examples
- Audio recording device
- For oral consent: record separately and store apart from the interview.
- For written consent: send in advance and request signed return before the interview.

### Interview Structure

Welcome & Introduction (10 min)

Part 1: Experience Supporting Pregnant Women (and Partners) in Decision-Making incl.

Recognizing and Working with the Target Group (30 min)

Part 2: Experience with Support Tools (15 min)

Part 3: Needs for the Intervention (30 min)

#### Welcome + introduction

- Welcome participant
- Introduce yourself (interviewer)
- Explain interview purpose: “TNO, Pharos, and the Midwifery Academy Maastricht are developing a training program for midwives to support pregnant women in prenatal screening decisions. This includes screening for Down, Edwards, and Patau syndromes, and the 20-week ultrasound. We are also developing a program for pregnant women, but this interview focuses on the program for you as a midwife.”
- Overview of interview topics: Experiences supporting pregnant women in decision-making, use of support materials, and needs & wishes for the new program.
- Explain recording and privacy: The interview takes approximately 90 minutes. It will be recorded and data will be anonymized. The recording will be deleted after summarizing all information. Participation is voluntary, and you may stop at any time. For participation start we require your informed consent. If you have any questions you may pose them anytime.
- Confirm consent (if online, already received).

#### Background Questions

Before we start I would like to ask some background questions.

-What is your age?

-At what practice are you currently working? (name, location)

-How many years have you been practicing as a midwife?

-How many years of experience do you have counseling on prenatal screening?

#### Part 1: Experience Supporting Pregnant Women (and Partners) in Decision-Making (30 min)

**Recognizing of, and experiences with the target group**

-Do you notice pregnant women struggling to understand prenatal screening information sometimes?

-What are the characteristics of these women, or couples? How do you recognize this group?

-Why, according to you, do these groups struggle with understanding the information?

-Which topics are difficult to understand?

-Which questions do you (regularly) receive?

-When does counselling on prenatal screening go well? Could you give some examples?

-How do you see your own role in this?

**Counseling Experiences**

-Do you sometimes struggle to explain information? With which topics?

-What challenges do you face (in counselling on prenatal screening)?

-How do you cope with those challenges (to support women with understanding the information and decision-making)?

-Could you recall some positive experiences in supporting pregnant women who have difficulties with understanding the information regarding prenatal screening? What works well so they can better understand and make a choice?

-Could you recall negative experiences? What doesn’t work?

-How do you prepare for the counseling on prenatal screening?

-How much time do you spend preparing?

-In how far do you check if information is understood? How?

-How much time do you spend supporting pregnant women who have difficulties with understanding information regarding prenatal screening? Do you feel you have enough time to support these women?

-How important is it to spend extra time and attention?

-To what extent do you feel equipped to discuss prenatal screening with women who struggle to understand information regarding prenatal screening (e.g., due to low literacy or language barriers)?

#### Part 2: Experience with Support Tools (15 min) (for women who need extra support)

-Do you use support materials for counselling on prenatal screening? (Which?)

-Which tools help you identify women who struggle to understand the information?

-In how far does this help you to identify these women? What works well and why?

-What are positive experiences with such support materials? Why?

-What are challenges or negative experiences have you had? Why?

-What do you miss? What do you need?

-Which materials do you to prepare yourself for the counselling with pregnant women who have difficulties to understand the information?

-In how far does this help you to better prepare for the counselling with these pregnant

women? Why (not)?

-What are positive experiences? What did work, and why?

-What are challenges you face(d)? What are negative experiences, and why?

What tools do you use to prepare for conversations with women needing extra support?

-What do you miss? What do you need?

-Which materials/tools do you use during the counselling with pregnant women who have difficulties to understand the information? Think of counselling-support materials to interpret risks, discuss norms and values, decision-making, actions and consequences, and check if information is understood.

- How do these tools help you support these women? Why (not)?

-To what extent does this help to stimulate pregnant women to ask personal questions?

-What are positive experiences? What did work, and why?

-What are challenges you face(d)? What are negative experiences, and why?

What tools do you use to prepare for conversations with women needing extra support?

-What are you missing? What do you need?

#### Part 3: Needs for the Intervention (30 min)

We are developing online materials for you to better support pregnant women who struggle with understanding information related to prenatal screening. These materials are meant for you to prepare for the consult, or to use during the consult. We’d like your input regarding needs and wishes.

I’ll show you some examples of potential program functionalities and would like to hear your first impression. The examples are not related to prenatal screening. Therefore try not to focus on the content, but on format and delivery.

##### Oudercoach: https://oudercoach.guidingtube.com/

[Show, let them experience the home page and 1 video (2 questions + feedback)].

-What is your first impression?

-What appeals to you and why?

-What doesn’t appeal to you and why?

-What rating would you give and why?

-Imagine we would make this for you so that you can support pregnant women who struggle with understanding information related to prenatal screening. In this case you would be faced with a challenging and are asked to choose an appropriate reaction, and receive feedback.

-In how far would you use this? Why (not)?

-What do you like or dislike?

##### 22-weeks vaccination: <https://tno-kinkhoest.aanzee.online/>

##### Prepare conversation

[View and let them use functionality “prepare conversation” section: [*https://tno-kinkhoest.aanzee.online/in-gesprek*](https://tno-kinkhoest.aanzee.online/in-gesprek)*]*

-What is your first impression?

-What appeals to you and why?

-What doesn’t appeal to you and why?

-What rating would you give and why?

-Imagine we would make this for you so that you can prepare for the conversation with pregnant women who struggle with understanding information related to prenatal screening.

-In how far would you use this? Why (not)?

-What do you like or dislike?

##### Knowledge Test

[View and let them use functionality “Knowledge test” section: [*https://tno-kinkhoest.aanzee.online/test-je-kennis*](https://tno-kinkhoest.aanzee.online/test-je-kennis)]

-What is your first impression?

-What appeals to you and why?

-What doesn’t appeal to you and why?

-What rating would you give and why?

-Imagine we would make this for you including questions regarding how to identify and support pregnant women who struggle with understanding information related to prenatal screening.

-In how far would you use this? Why (not)?

-What do you like or dislike?

##### Talent in Huis

[Show and let them go through beginning to getting to know the first person: https://www.talentinhuis.nl/]

-What is your first impression?

-What appeals to you and why?

-What doesn’t appeal to you and why?

-What rating would you give and why?

-Imagine we would make this to help you support pregnant women who struggle with understanding information related to prenatal screening. You will then engage in a conversation with a pregnant woman, as in the case of talent in-house, and be presented with a challenging situation. You will then be asked to choose an appropriate response and receive feedback on what is and is not helpful.

-In how far would you use this? Why (not)?

-What do you like or dislike?

#### General Questions

You saw some examples of (digital) tools.

-Which example appealed most to you and why?

-Which appealed least and why?

-What do you like better, and what do you like less?

-How would you use this for prenatal screening?

-What requirements should the program meet (think of content, time, format, features)?

-What barriers for use do you foresee?

-What format do you prefer (text, images, audio, video)? Why?

-How much time would you spend on the program to prepare for the counseling?

-How much time would you spend using it during consultations?

-How would you like to use it? For yourself to prepare? With the pregnant women during consultation? (before, during, after)?

-What are your needs/wishes and wishes for the tool for you to be able to better prepare for the counselling with pregnant women who have difficulties with understanding information about prenatal screening?

-What are your needs/wishes for the tool to support you in counselling and supporting women in decision-making?

-What topics must be included? Think of challenges you currently face it should help solve?

-Do you have any questions or tips not yet discussed?

#### Closing

-Thank participant

-Ask if they have questions

-provide gift card (or collect details to send if online)

-For questions or comments, contact: [researcher information]
