# Supplementary material for: Development of a Tailored Online Video-Based Assistant to Support Prenatal Screening Decisions in Couples With Limited Health Literacy: User-Centered Design Approach
Source: JMIR Form Res. 2026 Mar 27;10:e75391. doi: 10.2196/75391 (PMC13069372; doi:10.2196/75391)
Supplement: Multimedia Appendix 3 [file formative_v10i1e75391_app3.docx]

## Multimedia Appendix 3.

Screenshots of the decision aid

(1)
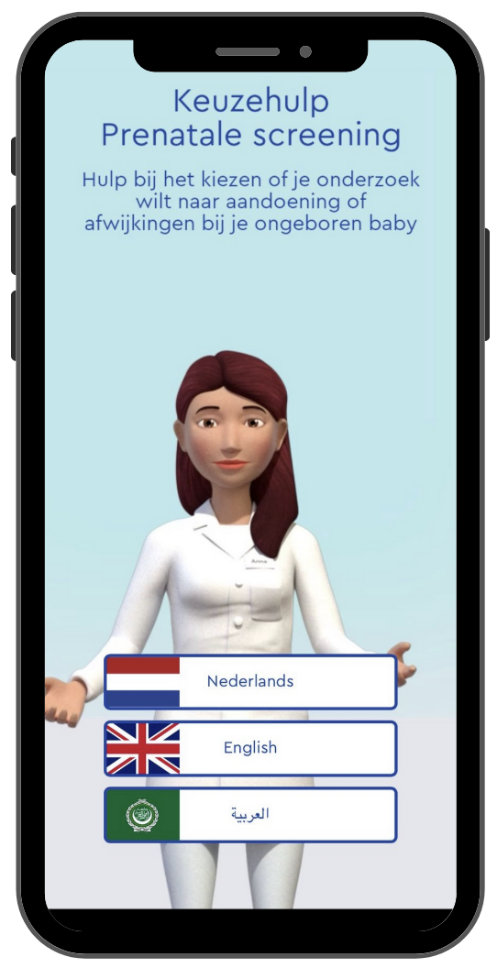
(2)
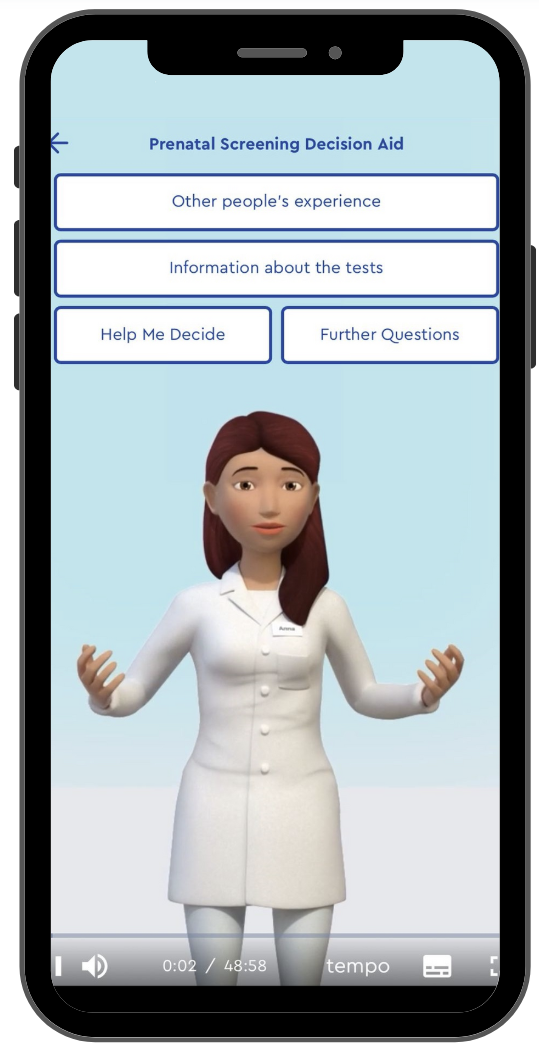
(3)
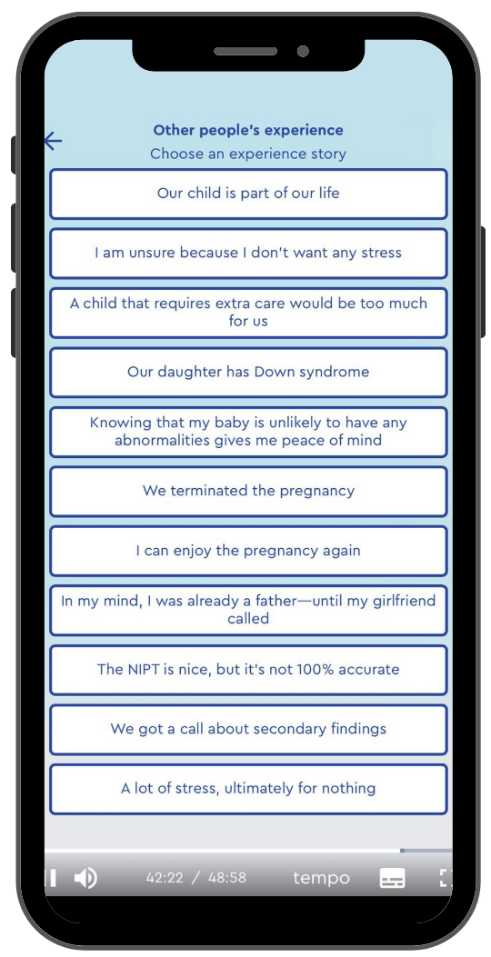
(4)
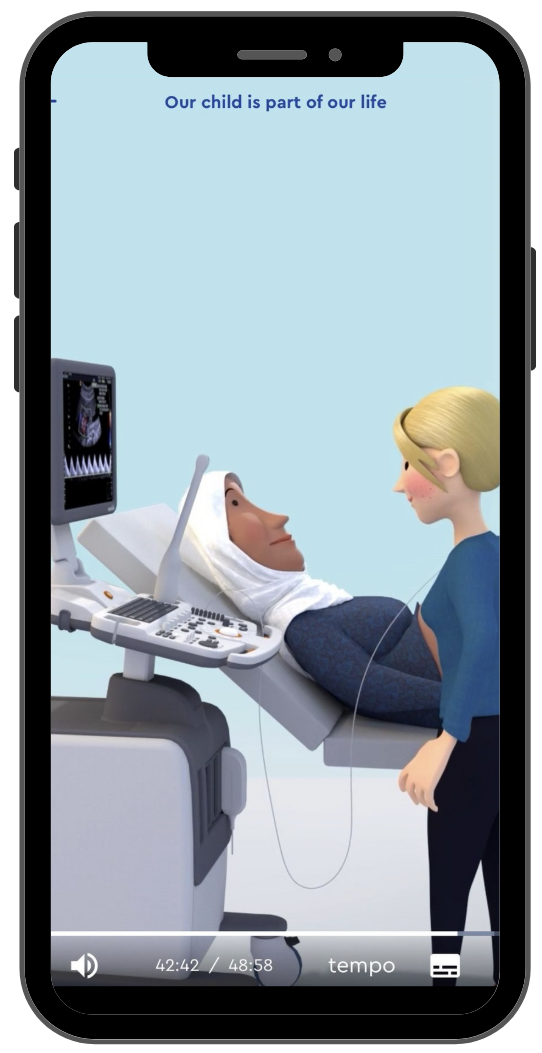


**Description:** (1) Home screen decision aid prenatal screening with choice options for various languages,

(2) Navigation menu with all four modules,

(3) Module ‘other people’s experience’ with scenarios to choose from,

(4) Audio-visual depiction of the scenario ‘Our child is part of our life’.

(5)
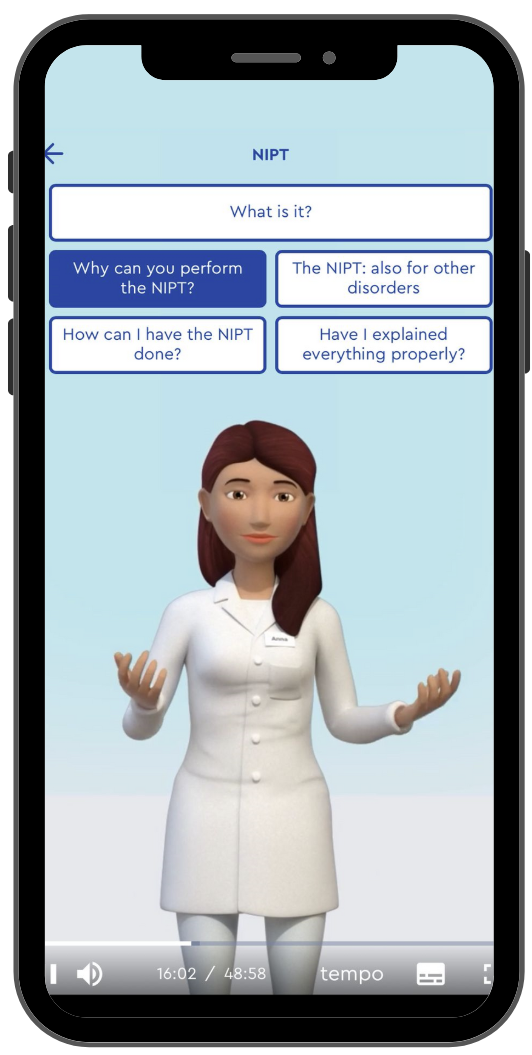
 (6)
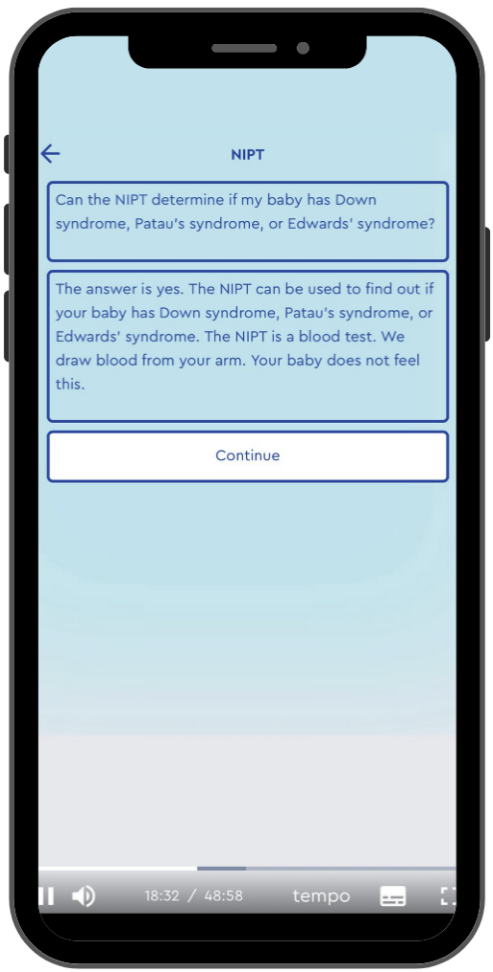
(7)
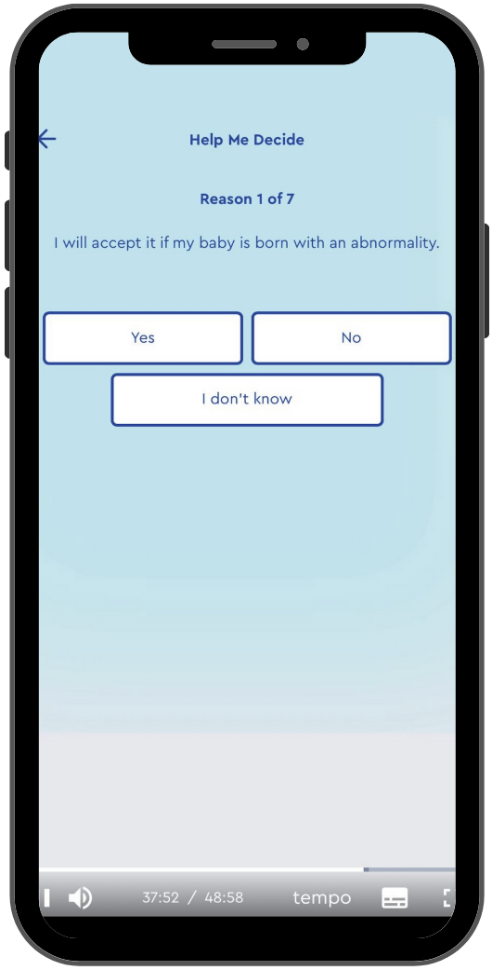
(8)
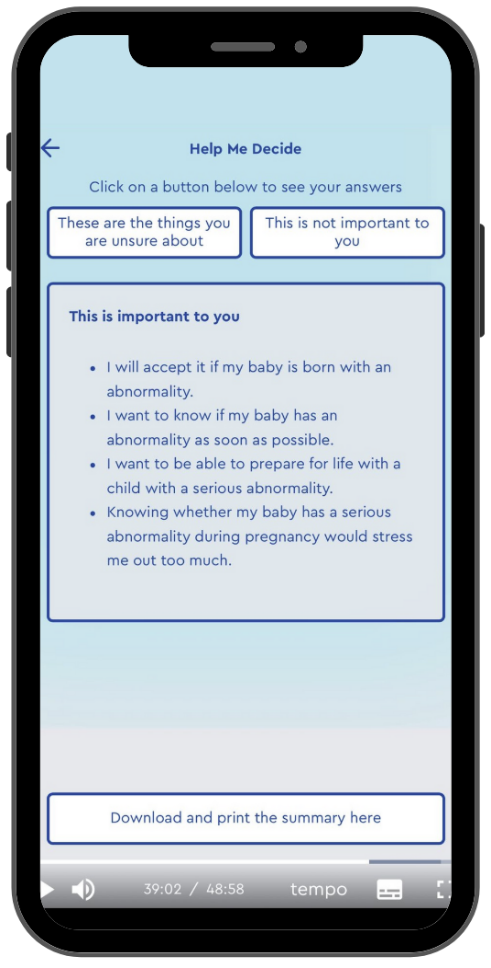


**Description**: (5) Example of information section “NIPT – what is it?” in the module “information about the tests”,

(6) Example of the teach-back question and revealed answer after user has clicked on “show answer”,

(7) Help me decide module example reason 1 of 7,

(8) Personal summary the user receives after indicating which reasons are important to them, the user can also choose to display reasons indicated as not important to them, and reasons they are usure about. The user is offered to download and print their summary.

(9)
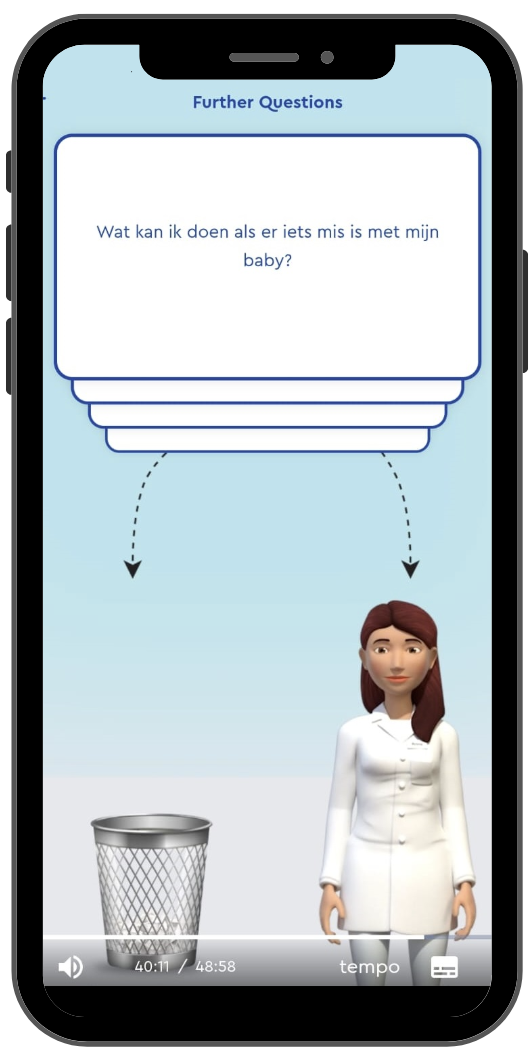
 (10)
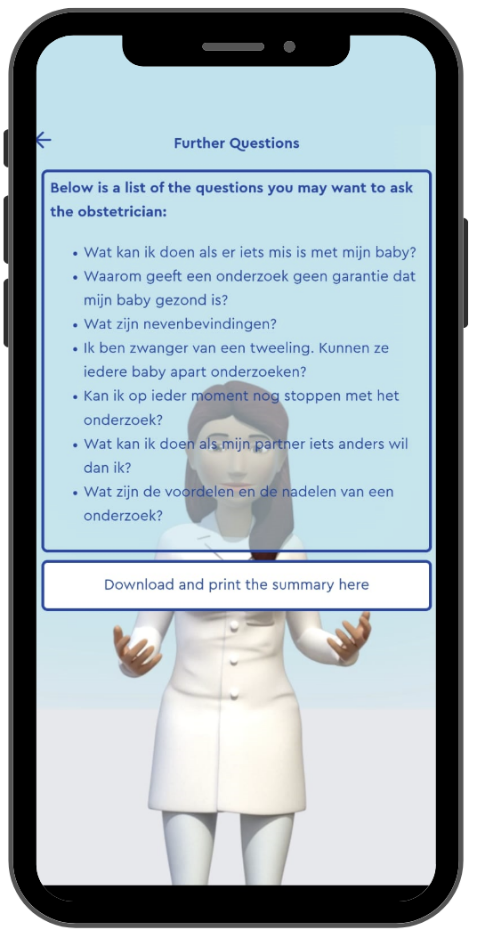
 (11)
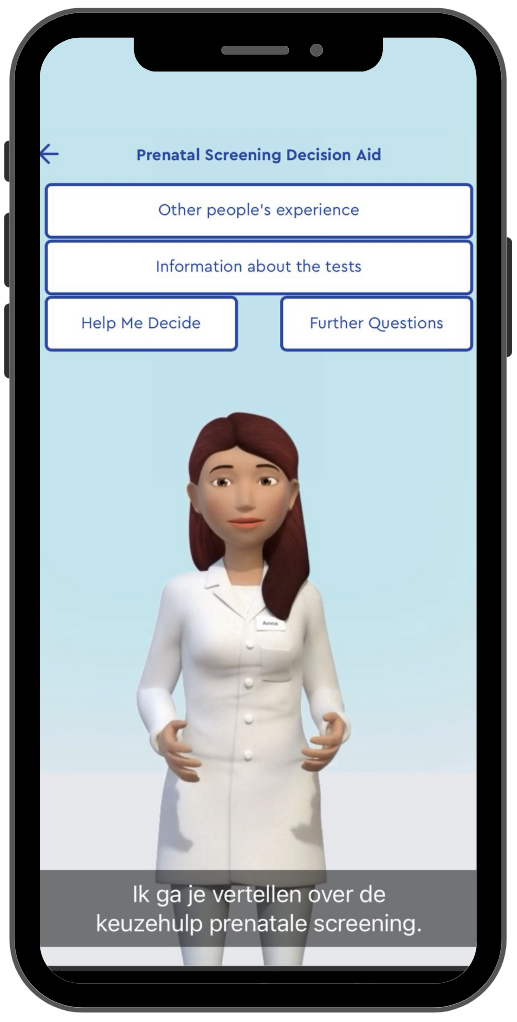
 (12)
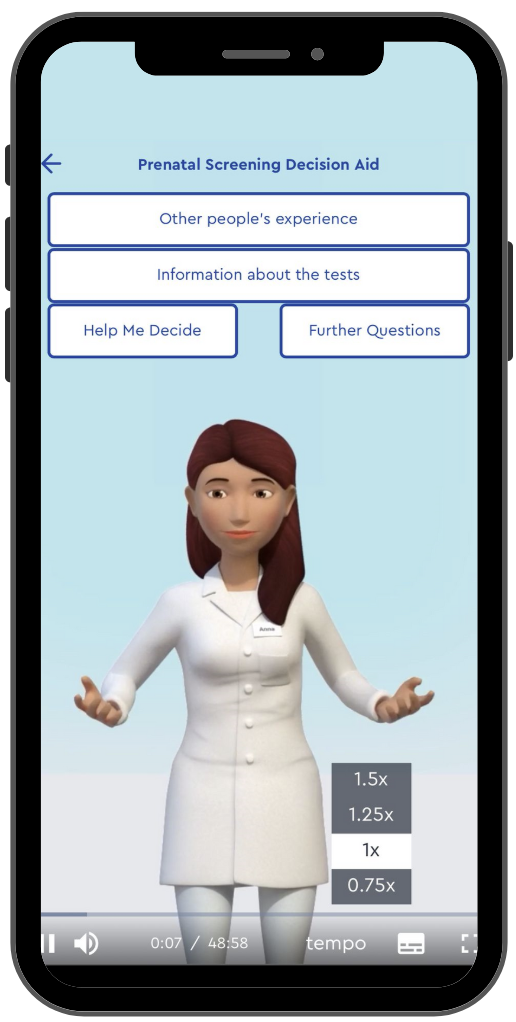


**Description:** (9) “Further questions” module (while the text is provided in Dutch, all questions and introductions are read aloud in English),

(10) Personal summary of remaining questions with the option to download and print the summary,

(11) Example of screen when Dutch subtitles are selected,

(12) Menu and options to adapt the speed of speech when button ‘tempo’ is selected in navigation pane (see yellow arrow).
