# Supplementary material for: Development of a Tailored Online Video-Based Assistant to Support Prenatal Screening Decisions in Couples With Limited Health Literacy: User-Centered Design Approach
Source: JMIR Form Res. 2026 Mar 27;10:e75391. doi: 10.2196/75391 (PMC13069372; doi:10.2196/75391)
Supplement: Multimedia Appendix 4 [file formative_v10i1e75391_app4.docx]

## Multimedia Appendix 4.

Table 3. Overview of modules and learning goals of the e-Learning.

| Module | Learning Goal | Content addressed/provided |
| --- | --- | --- |
|  |  |  |
| 1: Understanding low literacy better | Improve understanding of limited health literacy skills: Counsellors know why it is important to pay attention to low literacy among pregnant women during (prenatal screening) consultations. | Importance to have attention for pregnant individuals with limited health literacy skills in (prenatal screening) consults. |
|  |  | Difference between question demand or not-question demand examinations ((prenatal) screening belongs to the latter) |
|  |  | Importance of and definition of an informed choice is introduced, as well as what the options of pregnant individuals are in prenatal screening. |
|  |  | Fact chunks, such as ‘did you knows’ (e.g., that 25% of pregnant individuals does not make an informed decision about prenatal screening? They mostly decide what their sister/mother/friend/midwife would do). |
| 2: Recognizing signs of low literacy | Counsellors recognize signs of low literacy and know when pregnant couples have difficulty understanding information during (prenatal screening) consultations. | Importance to (1) check with every new pregnant individual whether limited health literacy skills exist and if so, (2) to note this in the midwifery practice files and (3) inform colleagues. |
|  |  | Questions to evaluate health literacy skills (e.g., “Many people have difficulty with reading brochures, how is that for you?”), and signals related to limited health literacy regarding reading, writing or telling a story (e.g., avoids reading or writing situations), regarding attention, questions or answers (e.g., reacts awkwardly when too many questions are posed), regarding keeping appointments or handing over papers (e.g., is too late to an appointment) and regarding medication (e.g., does not take medication properly). |
|  |  | More information: Pharos checklist for recognizing limited health literacy skills |
| 3: Communicating comprehensibly | Counsellors know what communication techniques to use with low-literate or pregnant couples who have difficulty understanding information during (prenatal screening) consultations. | Communication techniques to apply with pregnant couples with limited health literacy skills. It first provides tips about how to address limited health literacy respectfully, tips for use of language (use language of the individual, avoid imagery), the amount of information (summarize maximal three of the most important points) and for asking questions (e.g. which questions do you have instead of do you have questions?) |
| 4: Checking if information is understood | Counsellor knows how to check during consultations with pregnant couple to ensure that information about prenatal screening is understood. | Methods to check if information is understood, why it is important, how you can apply the methods (incl. examples). These are (1) the ‘teach back method’ (e.g., I would like to know if I have explained it well enough. What are you going to do or tell a significant other?), and (2) the ‘show-me method’ (e.g., I showed you where to find the decision aid. Can you show me how you will find it at home?). |
|  |  | Video to explain why it is important to check if the counselee understood the information. |
| 5: Using the prenatal screening decision aid as a tool | Counsellor knows what the prenatal screening decision aid is and how to use it during consultations with low-literate or pregnant couples who have difficulty understanding prenatal screening information. | How to apply the decision aid in prenatal screening (incl. the components of the decision aid with screen shots, navigation regarding language options and adjusting the speaking rate), and at what moments the decision aid can be applied (e.g., as a preparation to counseling or during counseling, before or during the prenatal screening contact moments like the 13-week echo, or in the waiting room. |
|  |  | Tips how to make individuals enthusiastic to use the decision aid, and how it can help them. |
